# Supplementary material for: Hybrid Scaffolds Decouple Biochemical & Biophysical Regulation of Cell Phenotype
Source: Adv Healthc Mater. 2025 Dec 7;15(9):e04086. doi: 10.1002/adhm.202504086 (PMC12973354; doi:10.1002/adhm.202504086)
Supplement: Supplementary file 1 — Supporting File: adhm70560‐sup‐0001‐SuppMat.docx. [file ADHM-15-0-s001.docx]

Supporting Information

**Hybrid Scaffolds Decouple Biochemical & Biophysical Regulation of Cell Phenotype**

*Xinyuan Song, Samantha C. Mitchell, Abbie N. Smart, William Hardiman, Daniel V. Bax, Ceri E. Staley, Catherine Probert, Pamela Collier, Marian Meakin, Alison A. Ritchie, Tania Mendonca, Amanda J. Wright, Victoria James, Anna M. Grabowska, Catherine L. R. Merry, Serena M. Best*, Ruth E. Cameron*, Jennifer C. Ashworth**


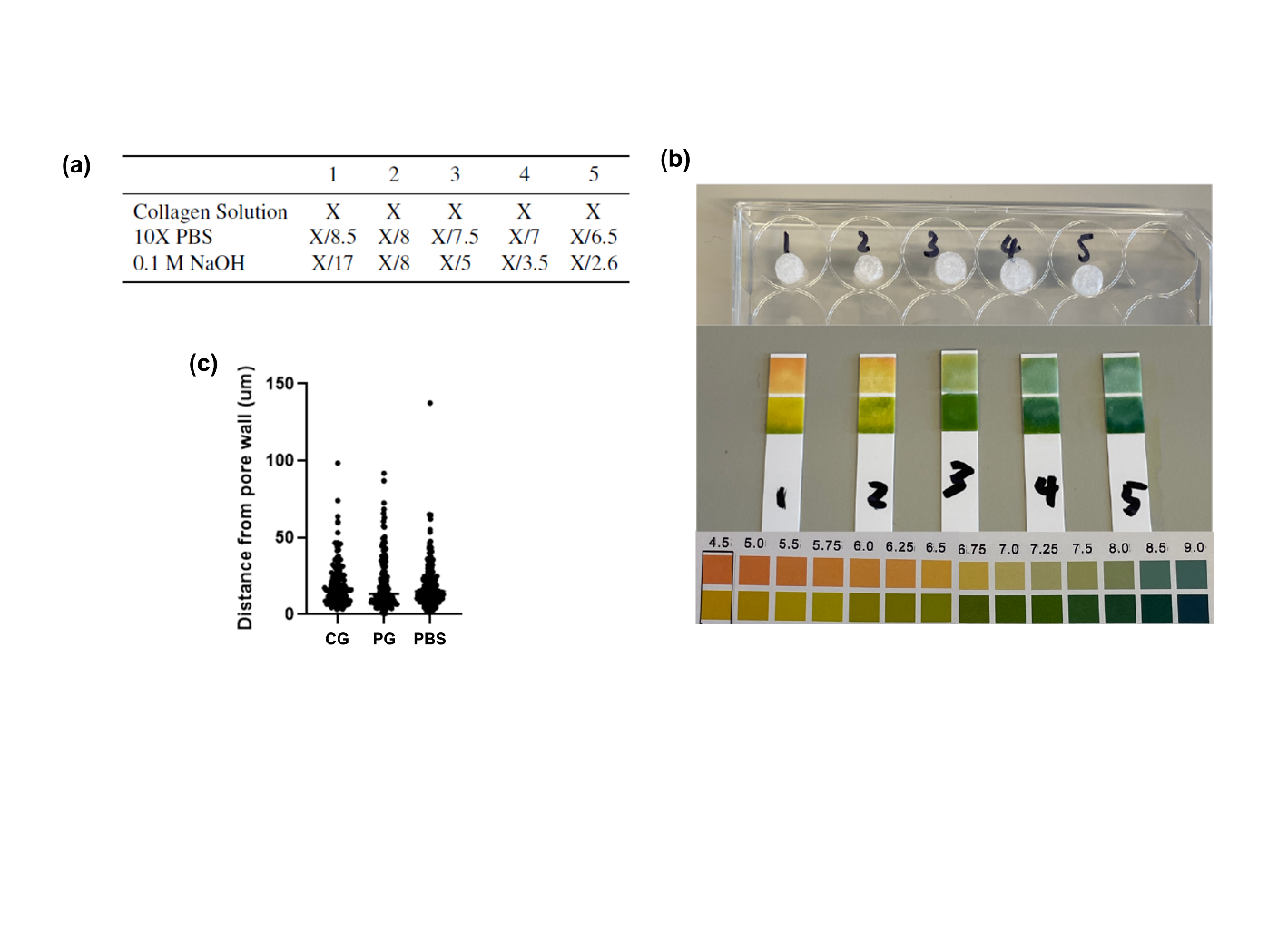


**Figure S1.** Optimisation and validation of hybrid scaffolds. (a) Table showing collagen gel formulations investigated for optimum gelation within hybrid scaffold; (b) pH measurements demonstrating the need for additional NaOH for collagen neutralisation in hybrid scaffolds relative to published protocols (formulation 1); (c) Quantification of bead distribution within hybrid scaffolds shows that both collagen gels (CG) and peptide gels (PG) penetrate effectively into the pore structure, showing no difference in bead positioning to a bead suspension in PBS alone.

**Figure S2.** Hybrid scaffolds containing collagen gels (Hy-CG) show no contraction over culture with human dermal fibroblasts (HDF). (a) Control measurements of water content in cell-free scaffolds incubated in PBS at 37°C overnight, demonstrating that all conditions have a water content of over 97% but that the collagen gel water content is significantly higher than the other tested conditions (p<0.0001, one-way ANOVA with Tukey post-hoc). (b) Measurements of (i) thickness and (ii) diameter of CG and Hy-CG with varying collagen concentration and % scaffold cross-linking (XL) reveal that contraction only occurs in CG alone, and not in Hy-CG. (c) Multiphoton images show that HDF cultured within Hy-CG have a rounded morphology across all collagen concentrations and cross-linking levels tested. Scale bar 200 µm.


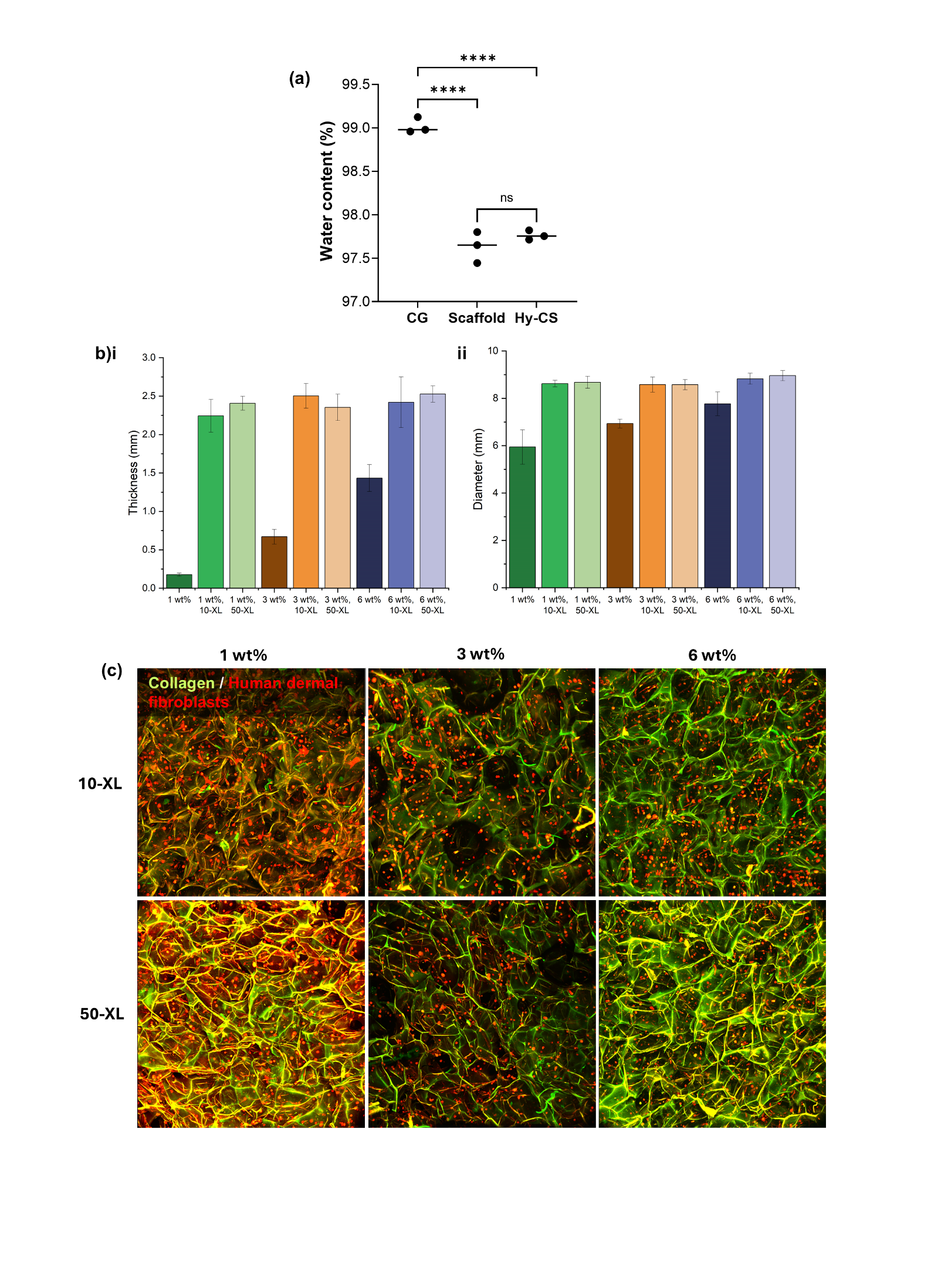


**Gel Scaffold Hy-CG**


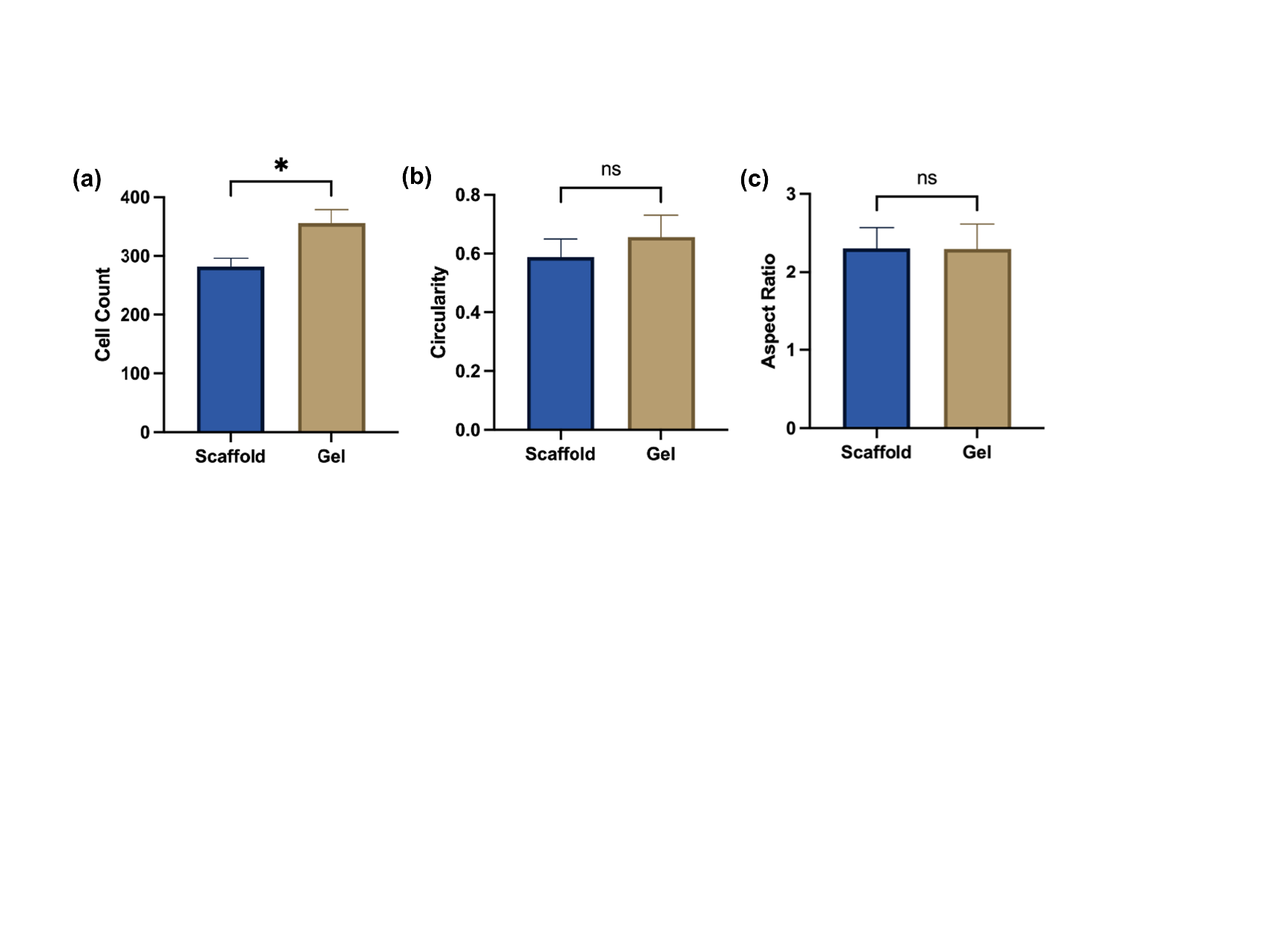


**Figure S3.** Cell seeding method does not affect human dermal fibroblast (HDF) morphology. (a-c) Comparison of cell count, circularity and aspect ratio of HDFs seeded into Hy-CG via the scaffold or the CG component (seeding methods A and B in Figure 1). Cells were cultured for 2 days.


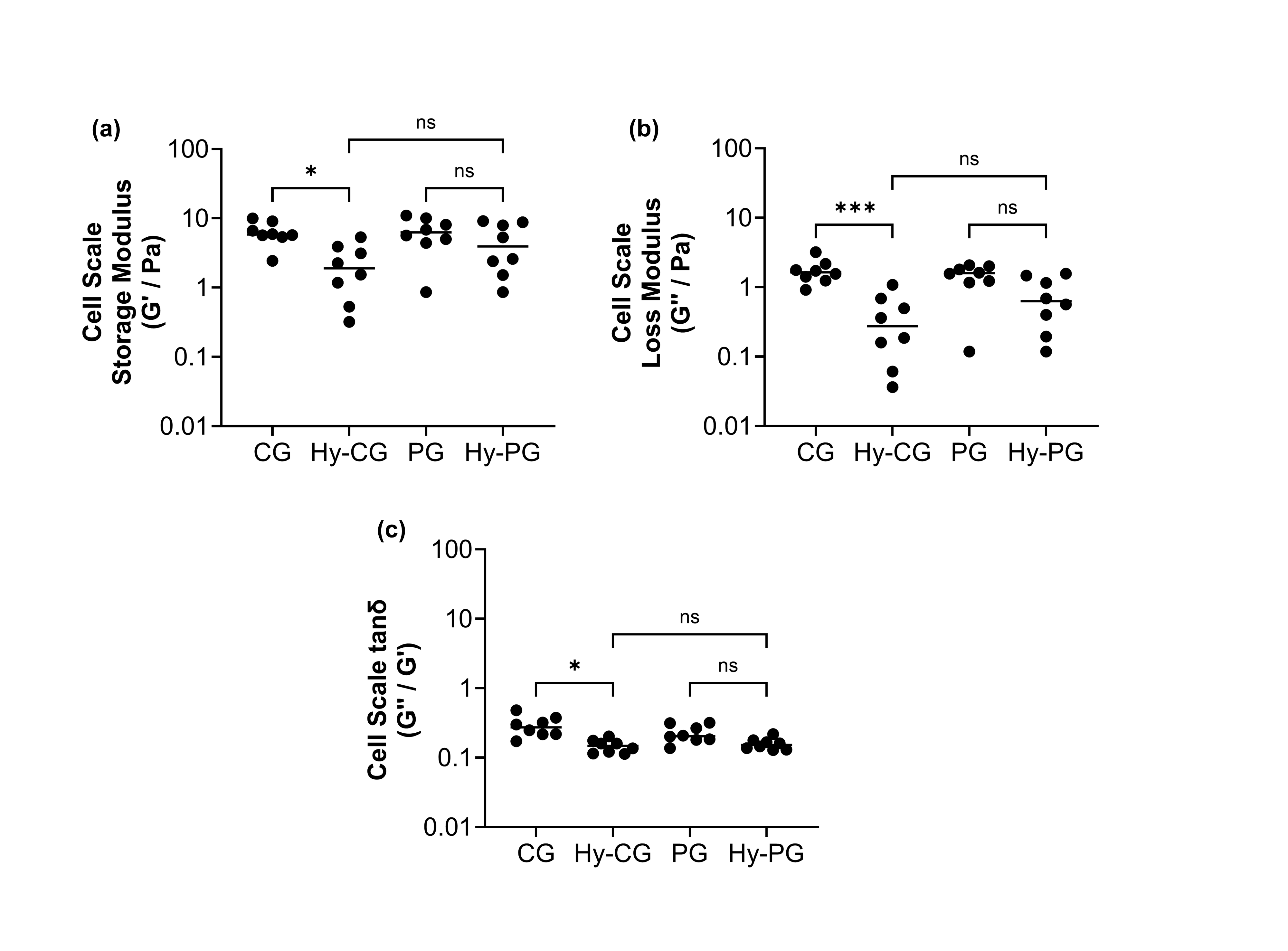


**Figure S4.** Local mechanical response differs between CG and Hy-CG but not between PG and Hy-PG. (a) Measurements of storage modulus *G’,* (b) Loss modulus *G’’* and (c) Loss tangent tanδ (*G’’/G’*) reveals no significant difference in any of these measurements between PG and the corresponding hybrid scaffold Hy-PG, in contrast to the comparison between CG and Hy-CG. Measurements for 8 beads from two samples per condition are shown.


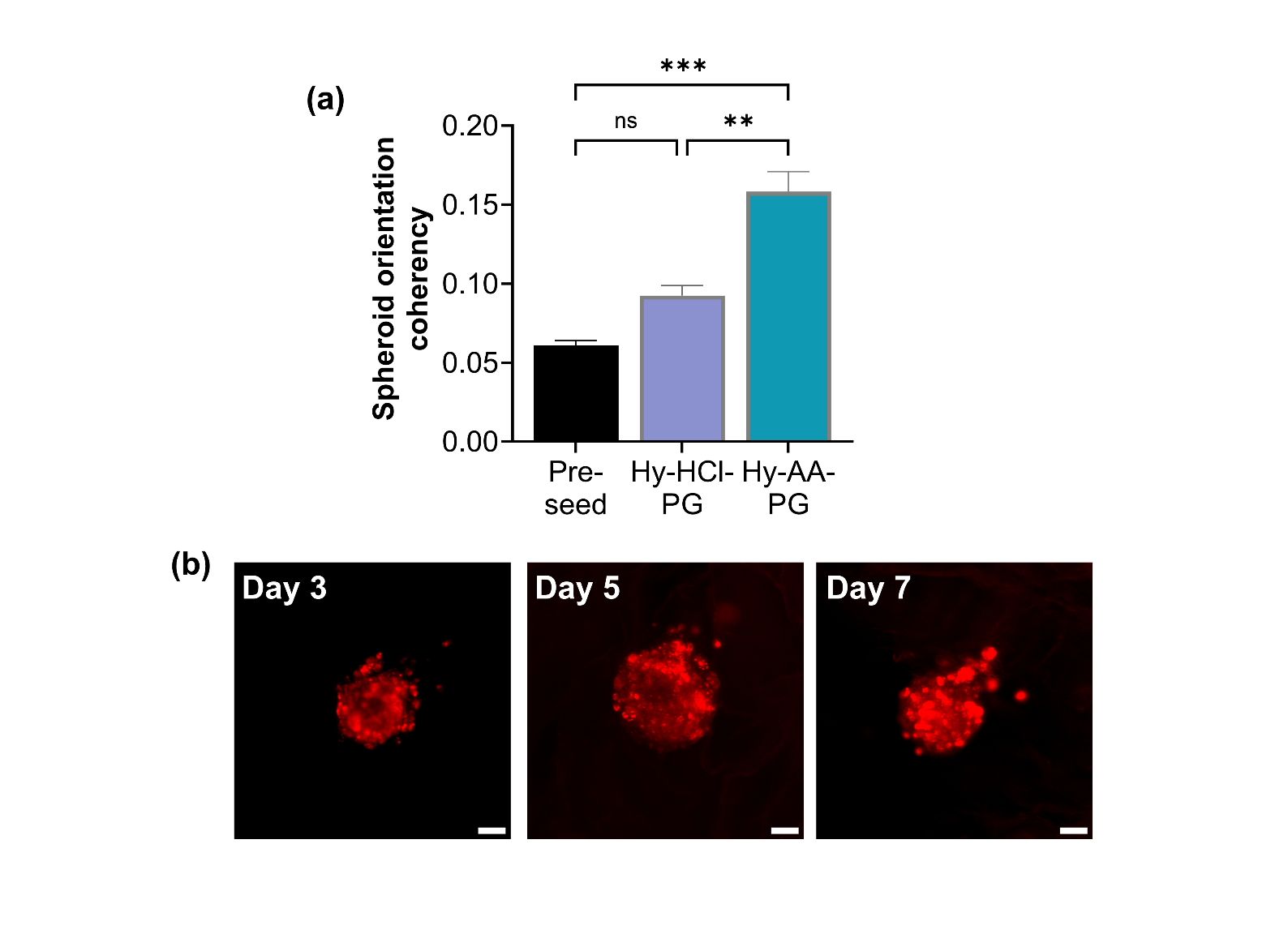


**Figure S5.** Hybrid scaffolds containing unmodified peptide gels direct breast cancer invasion. (a) Quantification of spheroid orientation coherency demonstrates significantly higher orientation coherency at constant spheroid size induced by the Hy-AA-PG hybrid scaffold relative to Hy-HCl-PG. Plots show mean and standard error of day 5 measurements from n=3 experimental repeats per condition, using PDX cells derived from at least 2 different patients; ** indicates p<0.01, *** indicates p<0.001. (b) Spheroid imaging demonstrates an increase in spheroid elongation over time in culture (images shown from Hy-AA-PG). Images have been adjusted to enhance spheroid visibility, since the intensity of the CellTracker dye reduces over time (note that this effect can make spheroids appear smaller at later time points as signal is lost). All scale bars 100 µm.
